# Four Complete Genome Sequences for *Bradyrhizobium* sp. Strains Isolated from an Endemic Australian *Acacia* Legume Reveal Structural Variation

**DOI:** 10.1128/MRA.00229-21

**Published:** 2021-05-13

**Authors:** Ming-Dao Chia, Anna K. Simonsen

**Affiliations:** a Research School of Biology, Australian National University, Canberra, Australian Capital Territory, Australia; b Land and Water, CSIRO, Canberra, Australian Capital Territory, Australia; c Department of Biological Sciences, Florida International University, Miami, Florida, USA; University of Arizona

## Abstract

*Bradyrhizobium* sp. strains were isolated from root nodules of the Australian legume, Acacia acuminata (Fabaceae). Here, we report the complete genome sequences of four strains using a hybrid long- and short-read assembly approach. The genome sizes range between ∼7.1 Mbp and ∼8.1 Mbp, each with one single circular chromosome. Whole-genome alignments show extensive structural rearrangement.

## ANNOUNCEMENT

Diazotrophs in the genus *Bradyrhizobium* (*Bradyrhizobiaceae*) are common and widespread root symbionts of many legume species worldwide. Within Australia, *Acacia* (Fabaceae) is a highly diverse and functionally important legume genus, and its symbiosis with *Bradyrhizobium* provides critical ecosystem services to native Australian vegetation (1–4).

Here, we report 4 complete *Bradyrhizobium* sp. genome sequences originally isolated from Acacia acuminata (a host endemic to southwest Australia, a global biodiversity hot spot). These genome sequences were completed to provide preliminary insight into the chromosome structural variation; the strains sequenced were selected to maximize genetic variability from a larger population genomic study comprising 375 closely related yet genetically diverse *Bradyrhizobium* strains sampled along a large climate gradient in the same region (5). All 4 *Bradyrhizobium* strains cluster within a single species, having >99.5% 16S rRNA sequence identity, and phylogenetically cluster with Bradyrhizobium diazoefficiens (5).

All strains were grown on yeast extract mannitol plates from frozen stock cultures (70% glycerol, −80°C), previously isolated from root nodules of *Acacia acuminata* (3, 5). DNA was extracted from a single colony using a modified MoBio Ultraclean microbial isolation protocol, where cells were heat treated (60°C, 5 min) in lysis buffer prior to mechanical lysing. For genome assembly, we used a hybrid approach using short Illumina reads and long reads from either the PacBio or Nanopore sequencing platform. Short-read whole-genome paired-end 150-bp Illumina data were generated on two HiSeq 2000 lanes using Illumina Nextera XT library kits, following standard Illumina protocols (5), and trimmed using Trimmomatic v0.36 (6) (ILLUMINACLIP:adapters.fasta:2:30:10 LEADING:5 TRAILING:5 SLIDINGWINDOW:4:20 MINLEN:100). In 3 of 4 strains, long-read data were generated on a PacBio RS II system at the Macrogen sequencing facilities in South Korea; SMRTbell libraries were created using the protocol “Procedure and Checklist—10 kb Template Preparation and Sequencing (with Low-Input DNA)” (7), and each strain was sequenced on two single-molecule real-time (SMRT) cells. Long reads for the fourth strain were generated in-house at Research School of Biology labs, Australian National University. In brief, DNA was isolated using the high-molecular-weight method of Schalamun et al. (8) (excluding the chloroform cleanup). Unsheared DNA extract was then prepared using the Oxford Nanopore library kit (SQK-LSK108) and sequenced on one R9.4 FLO-MIN106 flow cell. The reads were base called using Guppy v3.0.3.

**FIG 1:**
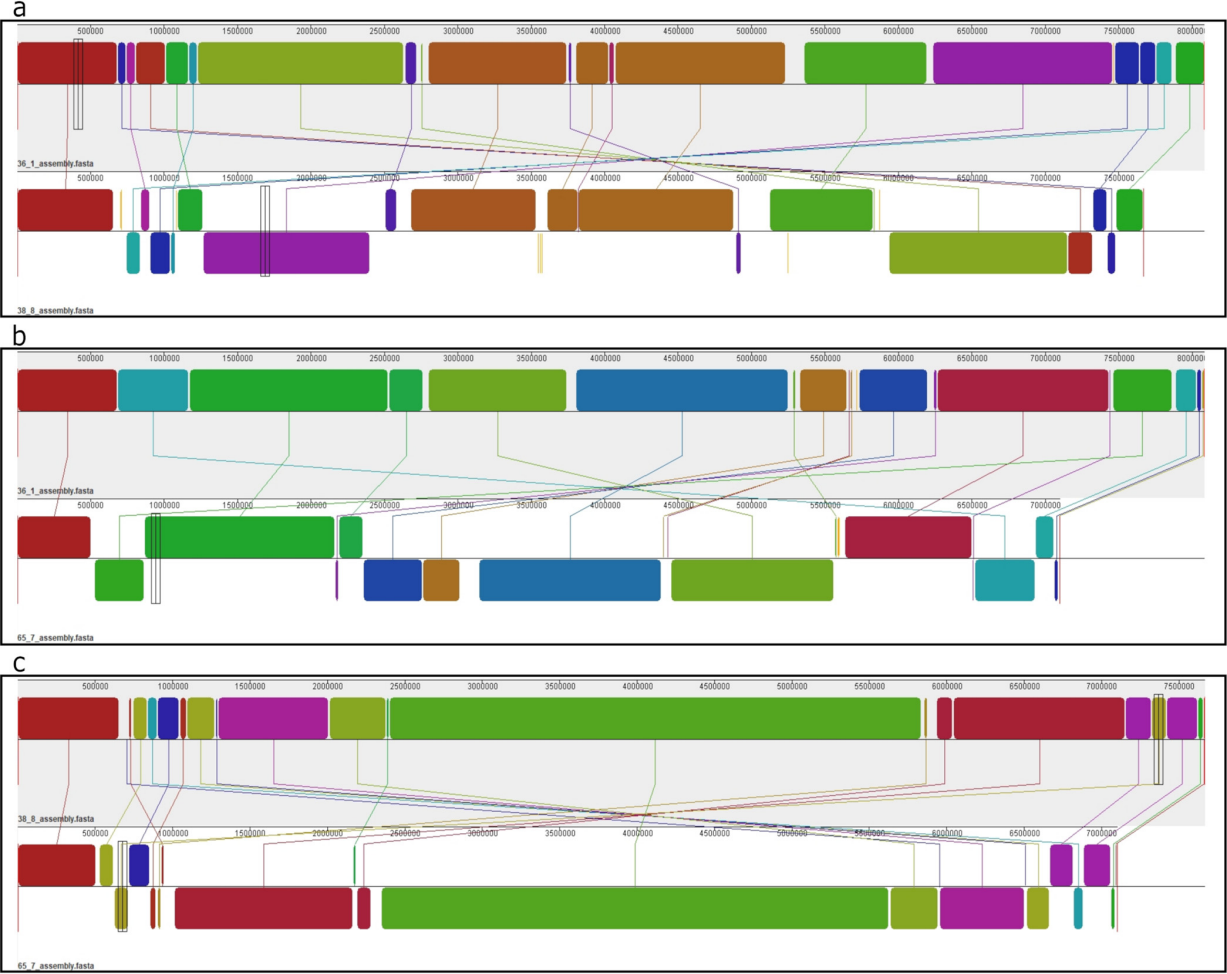
Pairwise whole-genome Mauve alignment output confirms the presence of large structural variation among circular chromosomes. Comparisons between strains where starting genes could be identified (36_1, 38_8, and 65_7; genome lengths in base pairs) are shown. For each comparison (a, b, and c), matching colored blocks and connecting lines indicate homologous genome sections between each pair. Inversions are indicated in the bottom genome of each pair (inversions are represented as matching color blocks below the black line). For example, one inverted genomic segment is visible between the matching purple blocks in panel a and the matching brown blocks in panel b. To facilitate visualization of the larger chromosomal rearrangements, the Mauve LCB weights (which adjust the single nucleotide polymorphism [SNP] similarity threshold) are adjusted to 13,166 (a), 12,932 (b), and 6,985 (c) for each pairwise comparison. The fourth strain (41_2) is not included in the comparison since a starting gene could not be identified, and it would visually indicate some false genomic rearrangements in Mauve’s linear chromosome alignment tool if included.

All genomes were assembled using Unicycler v0.4.8 with default settings (9). With the long and short reads combined, the total sequence data generated for each strain exceeded 113× coverage across each genome. The genome size, GC content, and gene number varied across strains (Table 1), but all strains contained one single chromosome and were designated with a complete circular status according to Unicycler. Starting genes were found for strains 65_7, 38_8, and 36_1, and the circular contigs were rotated accordingly, with the starting gene at the beginning of the forward strand. Unicycler did not find starting genes on strain 41_2. All genomes were annotated using NCBI’s PGAP v5.0 with default settings (10). Whole-genome alignments, using progressiveMauve v2.4.0 with default settings (11), confirm large structural rearrangements (Fig. 1) among the strains.

**TABLE 1:** Summary of the sequencing and genome assembly details for each strain

Strain	GenBank accession no.	Data for Illumina reads:		Data for long reads:					Genome size (bp)	Estimated coverage (×)	GC content (%)	Total no. of genes
		No. of reads	SRA accession no.	Platform	Library DNA input (μg)	No. of reads	SRA accession no.	*N*_50_ (bp)				
65_7	CP067041	8,512,491	SRR12822213, SRR12821956	PacBio	5	332,793	SRR12919153, SRR12919152	91,321	7,100,878	180	63.1	6,613
38_8	CP067100	6,105,011	SRR12822231, SRR12821976	PacBio	5	273,397	SRR12919157, SRR12919156	74,934	7,668,734	120	63.8	7,194
41_2	CP067101	5,913,600	SRR12822277, SRR12822021	PacBio	5	218,088	SRR12919155, SRR12919154	52,101	7,144,346	125	63.5	6,707
36_1	CP067102	6,115,736	SRR12822150, SRR12821895	Nanopore	1	242,922	SRR12919160	28,517	8,085,095	113	63.4	7,539

### Data availability.

The genome data are available in GenBank under BioProject accession number PRJNA669073 (SRA accession numbers are provided in Table 1). The Unicycler log files and Mauve alignment files are available on Figshare (https://doi.org/10.6084/m9.figshare.14134169).

## References

[1] Lafay B, Burdon JJ. 2001. Small-subunit rRNA genotyping of rhizobia nodulating Australian Acacia spp. Appl Environ Microbiol 67:396–402. doi:10.1128/AEM.67.1.396-402.2001. 11133471 PMC92591

[2] Rodríguez-Echeverría S, Crisóstomo JA, Freitas H. 2007. Genetic diversity of rhizobia associated with Acacia longifolia in two stages of invasion of coastal sand dunes. Appl Environ Microbiol 73:5066–5070. doi:10.1128/AEM.00613-07. 17545318 PMC1951011

[3] Dinnage R, Simonsen AK, Barrett LG, Cardillo M, Raisbeck-Brown N, Thrall PH, Prober SM. 2018. Larger plants promote a greater diversity of symbiotic nitrogen-fixing soil bacteria associated with an Australian endemic legume. J Ecol 107:977–991. doi:10.1111/1365-2745.13083.

[4] Le Roux C, Tentchev D, Prin Y, Goh D, Japarudin Y, Perrineau M-M, Duponnois R, Domergue O, de Lajudie P, Galiana A. 2009. Bradyrhizobia nodulating the Acacia mangium × A. auriculiformis interspecific hybrid are specific and differ from those associated with both parental species. Appl Environ Microbiol 75:7752–7759. doi:10.1128/AEM.01887-09. 19854923 PMC2794123

[5] Simonsen AK, Barrett LG, Thrall PH, Prober SM. 2019. Novel model-based clustering reveals ecologically differentiated bacterial genomes across a large climate gradient. Ecol Lett 22:2077–2086. doi:10.1111/ele.13389. 31612601

[6] Bolger AM, Lohse M, Usadel B. 2014. Trimmomatic: a flexible trimmer for Illumina sequence data. Bioinformatics 30:2114–2120. doi:10.1093/bioinformatics/btu170. 24695404 PMC4103590

[7] Pacific Biosciences. 2017. Procedure & checklist—10 kb template preparation and sequencing (with low-input DNA). Pacific Biosciences, Menlo Park, CA.

[8] Schalamun M, Nagar R, Kainer D, Beavan E, Eccles D, Rathjen JP, Lanfear R, Schwessinger B. 2019. Harnessing the MinION: an example of how to establish long-read sequencing in a laboratory using challenging plant tissue from Eucalyptus pauciflora. Mol Ecol Resour 19:77–89. doi:10.1111/1755-0998.12938. 30118581 PMC7380007

[9] Wick RR, Judd LM, Gorrie CL, Holt KE. 2017. Unicycler: resolving bacterial genome assemblies from short and long sequencing reads. PLoS Comput Biol 13:e1005595. doi:10.1371/journal.pcbi.1005595. 28594827 PMC5481147

[10] Tatusova T, DiCuccio M, Badretdin A, Chetvernin V, Nawrocki EP, Zaslavsky L, Lomsadze A, Pruitt KD, Borodovsky M, Ostell J. 2016. NCBI Prokaryotic Genome Annotation Pipeline. Nucleic Acids Res 44:6614–6624. doi:10.1093/nar/gkw569. 27342282 PMC5001611

[11] Darling AE, Mau B, Perna NT. 2010. progressiveMauve: multiple genome alignment with gene gain, loss and rearrangement. PLoS One 5:e11147. doi:10.1371/journal.pone.0011147. 20593022 PMC2892488

